# Study on Main Diffusion Coefficients and Atomic Mobility of Alloying Elements in the β-Phase of the Ti–Zr–Ta Ternary System

**DOI:** 10.3390/ma19071289

**Published:** 2026-03-24

**Authors:** Jingmin Liu, Danya Shen, Wenqing Zhao, Hongyu Zhang, Maohua Rong, Kaige Wang, Ligang Zhang, Libin Liu

**Affiliations:** 1 School of Materials Science and Engineering, Central South University, Changsha 410083, China; jingminliu24@163.com (J.L.); danyashen@163.com (D.S.); zhaowenqing2002@163.com (W.Z.); wangkaigek@163.com (K.W.); lbliu@csu.edu.cn (L.L.); 2 School of Materials Science and Engineering & Guangxi Key Laboratory of Information Materials, Guilin University of Electronic Technology, Guilin 541004, China; 3 Engineering Research Center of Electronic Information Materials and Devices, Ministry of Education, Guilin University of Electronic Technology, Guilin 541004, China; rongmh124@guet.edu.cn

**Keywords:** Ti–Zr–Ta alloys, numerical inverse method, main interdiffusion coefficients, atomic mobility

## Abstract

Diffusion-controlled processes exert an indispensable influence on the thermal processing and microstructural homogenization of *β*-titanium alloys containing multiple *β*-stabilizing elements. However, credible multicomponent diffusion kinetic data corresponding to the *β*-phase within the Ti–Zr–Ta ternary system remain inadequate. In this work, diffusion characteristics within the *β* single-phase domain of the Ti–Zr–Ta system were investigated using solid-state diffusion couples combined with a numerical inverse method. Twelve diffusion couples in total were synthesized and subjected to annealing treatments at 1373, 1423, and 1473 K, with the corresponding composition–distance distributions quantified by electron probe microanalysis (EPMA). The composition-dependent main interdiffusion coefficients were measured via the numerical inverse method embedded in the HitDIC computational platform, while the atomic mobility parameters corresponding to the *β*-phase were refined to replicate the experimental concentration distributions and diffusion trajectories across the studied temperature and composition intervals. The results reveal pronounced temperature and composition dependence of the main interdiffusion coefficients, and the diffusion rate of Zr is faster than that of Ta in the *β* phase.

## 1. Introduction

Titanium alloys have become key structural materials in aerospace, chemical engineering, and biomedical fields due to extraordinary specific strength [1,2], desirable biocompatibility [3], as well as superior high-temperature endurance [4]. Recent studies [5,6] have shown that titanium alloys doped with non-toxic β-stabilizing elements including Mo, Ta, Zr, and Nb (with elastic modulus E = 55–80 GPa) not only have mechanical properties more matching human bones but also exhibit significantly better hot workability, corrosion resistance, and grain refinement ability than α+β alloys [7], providing directions for the development of new-generation biomedical implant materials. As a commonly accepted fact, recrystallization, phase transformation, and grain growth processes exert a considerable influence on the mechanical properties and microstructural characteristics of structural metallic materials [8,9,10], with diffusion effects acting as the primary control mechanism for these processes [11,12].

As a powerful analytical technique, CALculation of PHAse Diagram (CALPHAD) plays a vital role in interpreting and predicting phase interactions and microstructural development processes in multicomponent alloys [13]. In this context, Andersson et al. [14] proposed the introduction of atomic mobility parameters into the CALPHAD formalism for describing diffusion behaviors in such alloy systems. By integrating moving interface models with local thermodynamic equilibrium theory, researchers are able to realize precise simulations of phase transition processes and elemental diffusion within alloy systems, which paves the way for subsequent microstructure evolution simulation work. By combining diffusion couple experiments with the numerical inverse method, researchers are able to establish a dependable and efficient procedure for measuring multicomponent main interdiffusion coefficients across wide composition intervals using a small number of experimental trials. By integrating experimentally measured concentration profiles with numerical inverse methods, researchers can extract composition-dependent diffusion coefficients with high efficiency and accuracy. This approach has been successfully applied to various multicomponent alloy systems. When combined with thermodynamic descriptions, such methods enable the evaluation of kinetic parameters that can reproduce diffusion profiles and diffusion paths within a defined temperature and composition range.

Although β–Ti–Zr–Ta alloys are increasingly used in load-bearing biomedical implants and high-temperature aerospace components, critical gaps remain in their diffusion datasets: few studies have reported the diffusion behavior of Ti, Zr, and Ta across the full β-phase stability range. Existing diffusion data for β–Ti–Zr–Ta alloys are limited to low Ta concentrations (<5 at. %) or temperatures outside the β-phase field.

In this study, diffusion couple technology combined with high-throughput calculation methods was used to conduct a systematic investigation into the Ti–Zr–Ta alloys diffusion behaviors. The study is primarily aimed at achieving the subsequent goals: (i) Prepare 12 sets of single-phase diffusion couples corresponding to the BCC Ti–Zr–Ta alloy systems, annealed at temperatures of 1373 K/48 h, 1423 K/36 h, 1473 K/24 h, and obtain composition–distance curves through electron probe microanalysis (EPMA) technology; (ii) determine composition-dependent main diffusion coefficients by high-throughput numerical inverse method, conducting comparative verification with the diffusion coefficients derived from the Matano–Kirkaldy method, and establishing the atomic mobility parameters; (iii) plot the temperature- and composition-dependent contour maps of the frequency factor, activation energy, and diffusion coefficient.

The main interdiffusion coefficients determined in the present study improve the diffusion database of the β-phase Ti–Zr–Ta alloy within the composition ranges of 0–16 at. % Zr and 0–16 at. % Ta at 1373–1473 K and provide reliable experimental data support for the subsequent fundamental research on the diffusion behavior of this system.

## 2. Experimental Procedures

Based on the thermodynamic descriptions of the Ti–Zr, Ti–Ta, and Zr–Ta binary systems provided by Turchanin, Barzilai, Fernández et al. [15,16,17], it can be predicted that a wide BCC_A2 single-phase region exists in the Ti-rich corner at the isothermal temperatures of 1373 K, 1423 K, and 1473 K.

On the basis of this finding, this study fabricated 12 distinct groups of diffusion couples for subsequent testing (Table 1), with end-member alloy compositions covering this single-phase region. The compositions of the selected diffusion couples are distributed as widely as possible within the β-phase region, so that the experimental data are more representative. In addition, the four diffusion couples at the same temperature provide exactly four intersection points, which satisfy the requirement for calculating diffusion coefficients at these intersections using the subsequent experimental method. The raw materials used were 99.99% purity Ti, Zr, and Ta metals, which were smelted six times in an argon-protected vacuum furnace to form homogeneous alloy samples. Subsequently, these specimens were encapsulated in quartz ampoules and subjected to annealing treatment at 1473 K for 168 h, with the aim of relieving casting-induced stresses and regulating grain dimensions to the millimeter range, minimizing interference from grain boundary diffusion and ensuring that lattice diffusion dominates. Each sample was precisely cut into 10 mm × 10 mm × 4 mm dimensions. The sample surfaces were subsequently ground and polished using SiC sandpaper, after which the diffusion couples required for the experiment were prepared by pressing. These couples were then placed in a tube furnace, heated to 1173 K and held for 4 h. They were subsequently sealed in vacuum quartz tubes for diffusion annealing: 1373 K/48 h, 1423 K/36 h, 1473 K/24 h. Subsequently, water quenching was performed immediately at a cooling rate exceeding 100 °C/s to freeze the diffusion microstructure (β phase) retained at high temperatures [18]. To mitigate oxidation and contamination, the diffusion couples were sealed in vacuum conditions. The interface cross-section of the diffusion couple was ground and polished prior to the collection of Ti, Zr and Ta concentration distribution data along the diffusion direction, which was accomplished with an electron probe microanalysis (EPMA, JEOL, JXA-8230, Tokyo, Japan). Line scans perpendicular to the diffusion interface were performed with a step size of 19.6 μm, a beam current of 10 μA, and a dwell time of 80 s per point. To ensure data consistency, the same acquisition parameters were adopted for all diffusion couples. Calibration was carried out using certified reference materials (CRMs) of Ti (99.99%), Zr (99.99%), and Ta (99.99%) to eliminate systematic errors, and the calibration error was verified to be less than 0.5%. Repeatability was validated by two approaches: (i) Four repeated scans on the same homogeneous sample, yielding an average relative standard deviation (RSD) of 3% for concentration measurements; (ii) three replicate diffusion couples, showing that the RSD of concentration measured at the same position was ≤3.2%. The average relative uncertainty of EPMA concentration measurements was determined to be 3%.

To ensure the reproducibility of the numerical inverse fitting process via the HitDIC (version V2.0) software, this study specifies the detailed settings of the core input parameters and boundary conditions adopted for the fitting. The input parameters include the original EPMA-measured composition–distance data of 12 diffusion couples (with a step size of 19.6 μm, beam current of 10 μA, and dwell time of 80 s per point) and the experimental actual temperature–time process parameters (1373 K/48 h, 1423 K/36 h, 1473 K/24 h). The end-member and binary terms of the thermodynamic factor and atomic mobility were fixed to the calibrated values reported in the literature [19,20,21,22] (the initial value of the ternary term was set to 0 as the parameter to be optimized), and the weights of the objective function were set as Wc = Wj = 0.5 and kept constant throughout the process.

Four physical constraints are also applied as boundary conditions during the fitting process: (i) Vacancy flux effect: Since Ti, Zr, and Ta have small differences in atomic radius and atomic mass, and all experimental temperatures lie within the stable β-phase region where diffusion is dominated by bulk diffusion, the vacancy flux effect is negligible. Therefore, the vacancy flux parameter is set to s = 0, and the vacancy flux correction is disabled. (ii) Diffusion stability constraint: To ensure physically meaningful interdiffusion coefficients and avoid unphysical fitting results, the thermodynamic stability criteria of the interdiffusion coefficient matrix are strictly enforced. Real-time verification and automatic parameter correction are performed during the iteration. (iii) Concentration and parameter feasible domain constraint: The actual compositions of the diffusion couple end-members are used as concentration boundaries without extrapolation or modification. The feasible domain of the ternary interaction terms to be optimized is limited to ±5 × 10^5^ J·mol^−1^. (iv) Iteration convergence constraint: To balance fitting accuracy and computational efficiency while ensuring full convergence for the ternary system, the Levenberg–Marquardt algorithm is adopted, with a convergence threshold of 1 × 10^−6^ and a maximum number of iterations of 5000.

Independent researchers can reproduce the fitting process without additional assumptions or post-processing by using HitDIC version V2.0 or above, selecting the multicomponent alloy diffusion couple fitting module (with phase transformation and grain boundary diffusion corrections disabled), inputting the original data in the format of distance (m)—atomic fraction of Ti/Zr/Ta, and assigning values to the parameters and constraints according to the above settings.

## 3. Experimental Methods

There are two methods commonly used to calculate the diffusion coefficients, namely the Matano–Kirkaldy method and the numerical inverse method [23].

### 3.1. Matano–Kirkaldy Method

For the measurement of ternary diffusion coefficients, the Matano–Kirkaldy method has been widely utilized in academic studies [24]. By introducing the Boltzmann parameter [25] and applying the initial conditions, the interdiffusion fluxes of the Ti–Zr–Ta ternary system can be described by the following expressions:

(1) $${\tilde{J}}_{Ta}=\frac{1}{2t}\int_{c_{Ta}^{-}\;or\;c_{Ta}^{+}}^{c_{Ta}}\left(x-x_{M}\right)dc_{Ta}=-{\tilde{D}}_{TaTa}^{Ti}\frac{{\partial c}_{Ta}}{\partial x}-{\tilde{D}}_{TaZr}^{Ti}\frac{\partial c_{Zr}}{\partial x}$$

(2) $${\tilde{J}}_{Zr}=\frac{1}{2t}\int_{c_{Zr}^{-}\;or\;c_{Zr}^{+}}^{c_{Zr}}\left(x-x_{M}\right)dc_{Zr}=-{\tilde{D}}_{ZrTa}^{Ti}\frac{{\partial c}_{Ta}}{\partial x}-{\tilde{D}}_{ZrZr}^{Ti}\frac{\partial c_{Zr}}{\partial x}$$

where x represents distance, $x_{M}$ represents the location of the Matano plane, t represents the diffusion time, $c_{i}$ represents the concentration of component i, while ${c_{i}}^{-}$ and ${c_{i}}^{+}$ correspond to the concentration values on the left and right sides of the diffusion couple, respectively. ${\tilde{D}}_{ZrZr}^{Ti}$ and ${\tilde{D}}_{TaTa}^{Ti}$ are defined as the primary diffusion coefficients, while ${\tilde{D}}_{ZrTa}^{Ti}$ and ${\tilde{D}}_{TaZr}^{Ti}$ represent the cross-diffusion coefficients, respectively.

Traditionally, four independent equations can be obtained through the construction of intersecting diffusion trajectories based on two diffusion couples, which facilitates the determination of primary diffusion coefficients (${\tilde{D}}_{TaTa}^{Ti}$, $\;{\tilde{D}}_{ZrZr}^{Ti}$) as well as cross-diffusion coefficients (${\tilde{D}}_{ZrTa}^{Ti}$, $\;{\tilde{D}}_{TaZr}^{Ti})\;$[26]. However, this method neglects the deviation of Matano planes among different elements. To address this issue, the normalized concentration parameter Yi was defined by Whittle and Green [24], with its specific expression shown below:

(3) $$Y_{i}=\frac{c_{i}-{c_{i}}^{-}}{{c_{i}}^{+}-{c_{i}}^{-}}$$

(4) $${\tilde{D}}_{TiTi}^{Ta}+{\tilde{D}}_{TiZr}^{Ta}\frac{dc_{Zr}}{{dc}_{Ti}}=\frac{1}{2t}\frac{dx}{dY_{Ti}}\left[\left(1-Y_{Ti}\right)\int_{-\infty }^{x}Y_{Ti}\cdot dx+Y_{Ti}\int_{x}^{+\infty }\left(1-Y_{Ti}\right)\cdot dx\right]$$

(5) $${\tilde{D}}_{ZrZr}^{Ta}+{\tilde{D}}_{ZrTi}^{Ta}\frac{dc_{Ti}}{{dc}_{Zr}}=\frac{1}{2t}\frac{dx}{dY_{Zr}}\left[\left(1-Y_{Zr}\right)\int_{-\infty }^{x}Y_{Zr}\cdot dx+Y_{Zr}\int_{x}^{+\infty }\left(1-Y_{Zr}\right)\cdot dx\right]$$

The modified equations enable accurate calculation of ternary interdiffusion coefficients (${\tilde{D}}_{TiTi}^{Ta}$, $\;{\tilde{D}}_{ZrZr}^{Ta}$, ${\tilde{D}}_{TiZr}^{Ta}$, ${\tilde{D}}_{ZrTi}^{Ta}$). Nevertheless, due to the low efficiency of this former approach, the numerical inverse method is more applicable to the high-throughput determination of ternary alloy diffusion coefficients.

### 3.2. Numerical Inverse Method

The aforementioned Matano–Kirkaldy method is applicable to calculating diffusion coefficients at the intersection of two diffusion paths, resulting in high computational cost and low efficiency despite its accuracy. To efficiently and accurately determine interdiffusion coefficients, Chen et al. [27] put forward the practical numerical inverse method in 2014. This method has been verified to enable efficient determination of the diffusion coefficients using only one set of diffusion couples [28]. According to the following principles:

Manning’s random alloy model describes the relationship between mobility $M_{i}$ and interdiffusion coefficients ${\tilde{D}}_{ij}^{3}\;\left(i,j=1\;or\;2\right)$ as follows [29]:

(6) $${\tilde{D}}_{ij}^{3}=RT\left(M_{i}\phi_{ij}^{3}-c_{i}\sum_{k}M_{k}\phi_{kj}^{3}\right)+s\left[M_{i}-\sum_{k}\left(c_{k}M_{k}\right)\right]\left[\frac{2c_{i}RT\sum_{k}\left(M_{k}\phi_{kj}^{3}\right)}{A_{0}\sum_{k}(c_{k}M_{k})}\right],\;(s=0)$$

Here R represents the gas constant, T represents absolute temperature, and $c_{i}$ represents the mole fraction of component i. Additionally, $\phi_{ij}^{3}$ denotes the thermodynamic factor. The second term represents the contribution of vacancy flow. Given the small differences in atomic radius and atomic mass among Ti, Zr and Ta, and the fact that all experimental temperatures in this study fall within the stable region of the β-phase where atomic diffusion is dominated by volume diffusion, s=0 is thus adopted here, indicating that the effect of the vacancy flow on the overall diffusion behavior is negligible. The expressions for atomic mobility parameters are as follows:

(7) $$\phi_{ij}^{3}=\frac{c_{i}}{RT}\left(\frac{\partial \mu_{i}}{{\partial c}_{j}}-\frac{\partial \mu_{i}}{{\partial c}_{3}}\right)$$

(8) $$M_{k}=\frac{1}{RT}\exp\left(\frac{∆G_{k}}{RT}\right)$$

where $\mu_{i}$ is the activity of component i. Studies have shown that minor differences in thermodynamic parameters have a negligible impact on calculated interdiffusion coefficients [30]. The polynomial expansion of Redlich–Kister for $∆G_{k}$ is expressed as follows:

(9) $$∆G_{k}=c_{1}{∆G}_{k}^{1}+c_{2}{∆G}_{k}^{2}+c_{3}{∆G}_{k}^{3}+c_{1}c_{2}{∆G}_{k}^{1,2}+c_{1}c_{3}{∆G}_{k}^{1,3}+c_{2}c_{3}{∆G}_{k}^{2,3}+c_{1}c_{2}c_{3}{∆G}_{k}^{1,2,3}$$

where ${∆G}_{k}^{j}(j$ = 1, 2, 3) denotes the end-member parameter corresponding to the diffusion of element k within element j, while ${∆G}_{k}^{i,j}(i$, j = 1, 2, 3) and ${∆G}_{k}^{1,2,3}$ represent binary and ternary interaction parameters, respectively, which are treated as optimization variables.

The numerical inverse method employs algorithms such as Levenberg–Marquardt [31] to iteratively minimize the error between experimental and calculated values until optimal atomic mobility parameters are obtained. The objective function is expressed as:

(10) $$min\langle error\rangle =min\langle W_{c}\sum_{i=1}^{N}\sum_{j=1}^{Num}\frac{\left(\left|c_{ij}^{cal}-c_{ij}^{exp}\right|\right)}{c_{ij}^{exp}}+W_{J}\sum_{i=1}^{N}\sum_{j=1}^{Num}\frac{\left(\left|{\tilde{j}}_{ij}^{cal}-{\tilde{j}}_{ij}^{exp}\right|\right)}{{\tilde{J}}_{ij}^{exp}}\rangle$$

where $W_{c}$ and $W_{J}$ are weights for concentration and diffusion flux errors, respectively, with default initial values of 0.5. $c_{ij}^{exp}$ and $c_{ij}^{cal}$ are experimental and calculated concentrations of element i at position j, while ${\tilde{j}}_{ij}^{exp}$ and ${\tilde{j}}_{ij}^{cal}$ are the corresponding interdiffusion fluxes. ${\tilde{j}}_{ij}^{cal}$ is calculated as:

(11) $${\tilde{J}}_{ij}^{cal}=-\sum_{k}^{2}{\tilde{D}}_{ik}^{3}\frac{\partial c_{j}}{\partial_{x}}\left(i=1,2\right)$$

Experimental interdiffusion fluxes at position x in composition–distance profiles are determined by:

(12) $${\tilde{J}}_{ij}^{exp}=\frac{c_{i}^{L}-c_{i}^{R}}{2t}\left[Y_{i}^{\prime }\int_{-\infty }^{X^{\prime }}\left(1-Y_{i}\right)dx+\left(1-Y_{i}^{\prime }\right)\int_{X^{\prime }}^{-\infty }Y_{i}dx\right]$$

By combining Equations (6) and (9), given initial values of optimization parameters and boundary conditions, composition–distance profiles can be simulated and compared with experimental data. Parameters are iteratively optimized according to Equation (10) until the minimal error is achieved, yielding the corresponding interdiffusion coefficients.

## 4. Results and Discussion

### 4.1. Diffusion Behavior of BCC_A2 Ti–Zr–Ta Ternary Alloys

**Figure 1:**
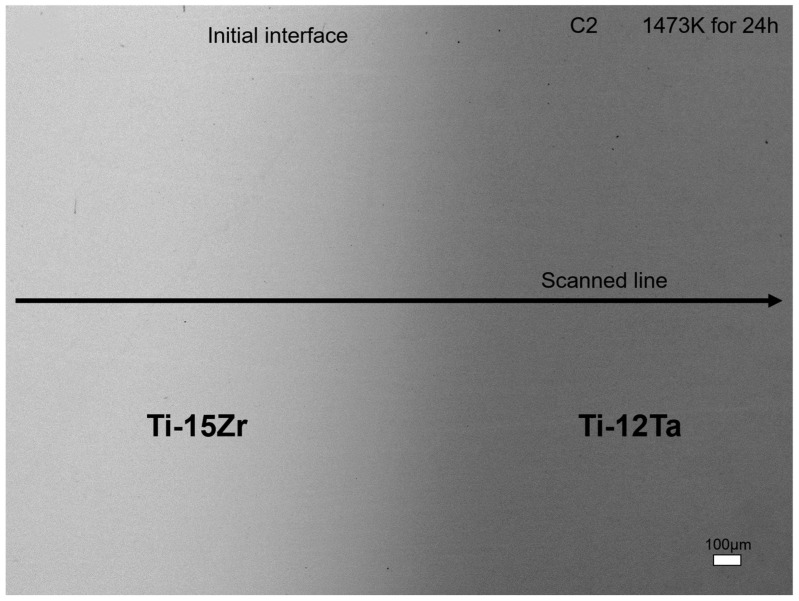
Backscattered electron image of diffusion couple C2 annealed at 1473 K for 24 h.

It can be clearly observed from Figure 1 that there is no obvious contrast in the two terminal regions of diffusion couple C2. The α−β phase transformation temperature of pure titanium is 883 °C [32], and the addition of the β-stabilizing element Ta further reduces this transformation temperature [33]. Therefore, the alloy microstructures are all single β-phase at 1373, 1423, and 1473 K. Meanwhile, the ultra-fast quenching rate adopted in this study is insufficient for diffusive phase transformations, so the single β-phase structure is retained. Although corresponding EBSD/XRD analysis was not performed in this study, it can be inferred that the diffusion couple maintains a stable β-phase at the aforementioned temperatures, and the employed annealing parameters can meet the experimental requirements for single-phase diffusion. These results not only verify the applicability of the BCC_A2 single-phase diffusion conditions but also lay a reliable microstructural foundation for the subsequent determination of elemental concentration distributions and calculation of interdiffusion coefficients via electron probe microanalysis (EPMA).

This study employs electron probe microanalysis (EPMA) to measure the composition–distance curves of ternary Ti–Zr–Ta diffusion couples. Leveraging the dedicated diffusion kinetics research software HitDIC, this study systematically analyzed the diffusion characteristics of BCC_A2-type Ti–Zr–Ta alloys by taking composition–distance data as input and invoking its uncertainty quantification functions, multivariate optimization algorithms, and an integrated advanced data processing module. The software enables the high-precision calculation of diffusion coefficients and facilitates parameter optimization and dynamic updating of atomic migration databases. The diffusivity function in this work is approximated using the thermodynamic characterization of the ideal solution phase coupled with atomic mobility parameters, which encompass end-member properties as well as binary and ternary interaction terms. To minimize discrepancies between the composition–distance profiles and the calculated diffusion coefficients, the aforementioned parameters were subjected to iterative optimization through the integration of a genetic algorithm and regularization techniques, with specific values summarized in Table 2.

**Figure 2:**
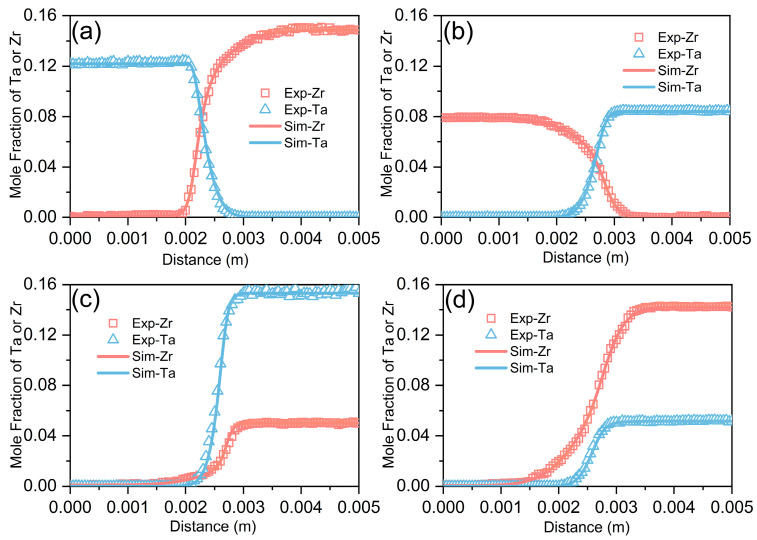
Composition–distance curves of diffusion couples (**a**) A1, (**b**) A2, (**c**) A3, (**d**) A4 subjected to 1373 K annealing for 48 h. Symbols represent the raw data, and the solid lines represent the software calculation results.

**Figure 3:**
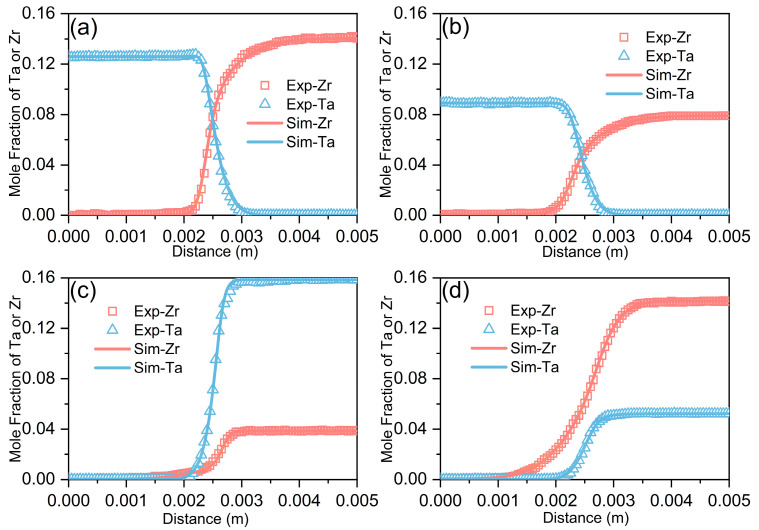
Composition–distance curves of diffusion couples (**a**) B1, (**b**) B2, (**c**) B3, (**d**) B4 subjected to 1423 K annealing for 36 h. Symbols represent the raw data, and the solid lines represent the software calculation results.

**Figure 4:**
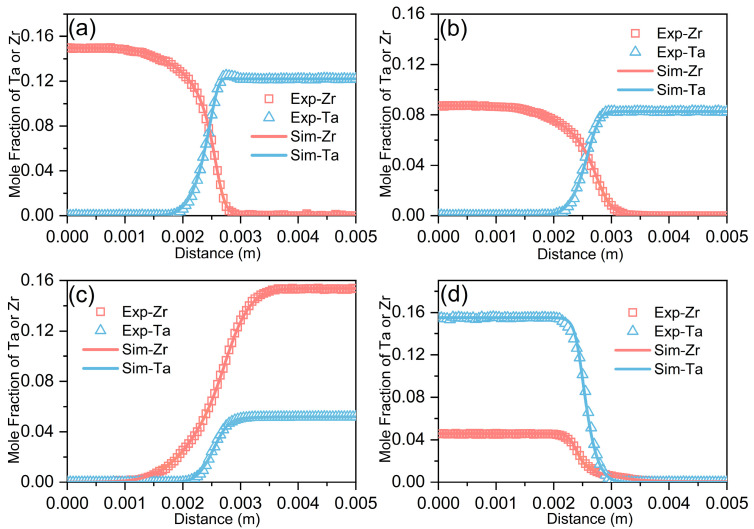
Composition–distance curves of diffusion couples (**a**) C1, (**b**) C2, (**c**) C4, (**d**) C3 subjected to 1473 K annealing for 24 h. Symbols represent the raw data, and the solid lines represent the software calculation results.

Figure 2, Figure 3 and Figure 4 illustrate the composition–distance profiles, and their fitted curves obtained from twelve sample sets measured at 1373 K, 1423 K, and 1473 K, respectively. Derived from the optimized atomic mobility database, the calculated results are depicted as solid lines, while experimental measurements are marked with triangular data points. The experimental curves show excellent consistency with the fitted curves. All diffusion couple combinations show obvious diffusion trends, and the diffusion distance is highly dependent on temperature. By comparing the curves of the same diffusion couple at different temperatures, it can be seen that all curves show uniform diffusion behavior, i.e., the diffusion rate increases significantly with increasing temperature (for example, the Zr diffusion distance in sample C3 at 1473 K is approximately 45% longer than that in sample A3 at 1373 K), even with shorter annealing times. Moreover, there is a consistently longer diffusion distance observed for Zr compared with Ta, revealing the intrinsic disparities in the diffusion capabilities of the respective components, and indicating that the former has a faster diffusion rate in the alloy system.

**Figure 5:**
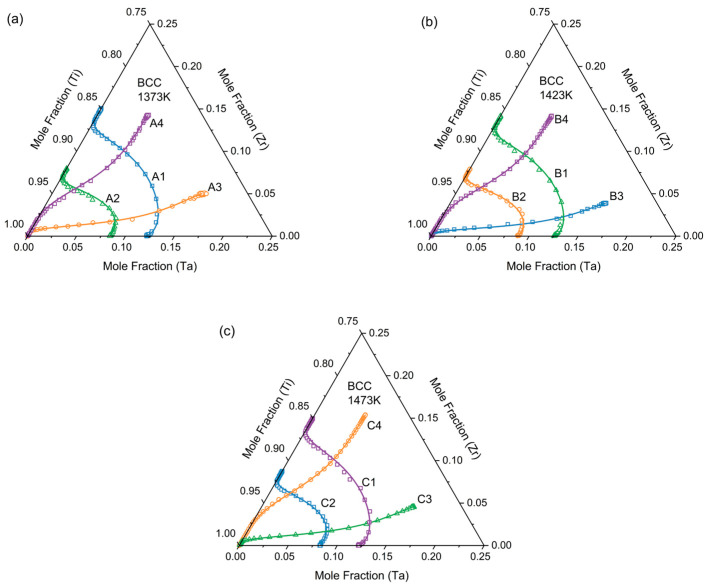
Model-predicted diffusion paths in the Ti–Zr–Ta system: (**a**) 1373 K for 48 h, (**b**) 1423 K for 36 h, (**c**) 1473 K for 24 h. Solid lines denote curves derived from the optimized atomic mobility database, and square or triangular symbols represent experimentally measured data.

Figure 5 presents the experimental and fitted curves of the Ti–Zr–Ta system in the BCC phase region of the isothermal sections at 1373 K, 1423 K, and 1473 K. All diffusion trajectories exhibit an S-shaped distribution in the figure, a typical signature of composition-dependent diffusion behavior, which indicates significant variations in diffusion coefficients with alloy composition.

### 4.2. Diffusion Coefficients of Ti–Zr–Ta BCC_A2

The theoretical framework elaborated in Section 3 is applicable for computing diffusion coefficients that are dependent on both composition and temperature. Within the scope of this research, Ti was designated as the dependent component for diffusivity annotation, on account of its capacity for equivalent variation [34]. As a consequence, four independent diffusion coefficients can be identified within the Ti–Zr–Ta system: ${\tilde{D}}_{TaTa}^{Ti}$, ${\tilde{D}}_{ZrZr}^{Ti}$, ${\tilde{D}}_{TaZr}^{Ti}$ and ${\tilde{D}}_{ZrTa}^{Ti}$. This study adopted the Matano–Kirkaldy method to calculate the diffusion coefficients of diffusion couples with intersecting diffusion paths, and the calculations satisfied the thermodynamic stability constraints [35].

(13) $${\tilde{D}}_{TaTa}^{Ti}+{\tilde{D}}_{ZrZr}^{Ti}>0$$

(14) $${\tilde{D}}_{TaTa}^{Ti}{\tilde{D}}_{ZrZr}^{Ti}-{\tilde{D}}_{TaZr}^{Ti}{\tilde{D}}_{ZrTa}^{Ti}\geq 0$$

(15) $${{(\tilde{D}}_{TaTa}^{Ti}-{\tilde{D}}_{ZrZr}^{Ti})}^{2}+4{\tilde{D}}_{TaZr}^{Ti}{\tilde{D}}_{ZrTa}^{Ti}\geq 0$$

**Figure 6:**
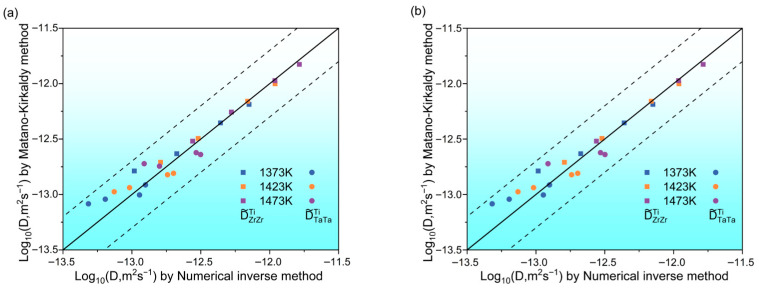
Comparison of main diffusion coefficients in BCC Ti–Zr–Ta alloys calculated using M-K method and numerical inverse method: (**a**) calculated by fitting using 12 datasets, (**b**) calculated by fitting using the first 11 datasets. The two dashed lines represent diffusion coefficients adjusted by factors of 2 and 0.5, respectively.

Using the EPMA concentration measurement uncertainty as input, uncertainty propagation was performed using the Monte Carlo method in the HITDIC software (1000 random samples). The 95% confidence interval of D is approximately ±6% over the entire composition range. For example, the 95% confidence interval of the maximum value of ${\tilde{D}}_{TaTa}^{Ti}$ at 1373 K is (2.04 ± 0.1224) × 10^−13^ m^2^/s. Figure 6 shows a comparison of diffusion coefficients obtained by two methods, where (a) is fitted using 12 datasets and (b) is fitted using the first 11 datasets. The solid diagonal line represents perfect agreement between the diffusion coefficients predicted by the model and those derived from the Matano–Kirkaldy method, while the dashed lines denote the coefficients scaled by a factor of 2 and 0.5, respectively. As shown in the figure, all data points fall within the acceptable error range, and the data points in (a) exhibit a slightly more concentrated trend than those in (b). Theoretically, a larger dataset is preferable, but the numerical inverse method can still maintain high accuracy even with a limited dataset [36], which verifies the reliability of the calculation procedure in this study.

**Figure 7:**
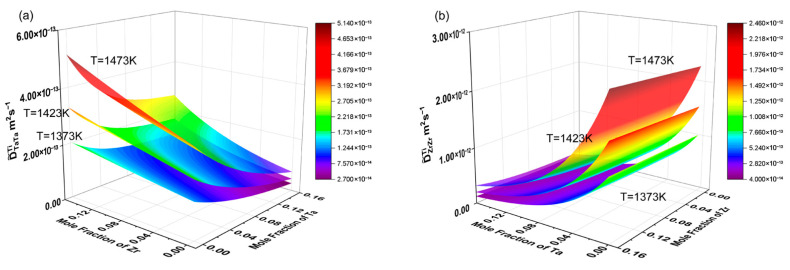
Three-dimensional surface plots of principal diffusion coefficients (**a**) ${\tilde{D}}_{TaTa}^{Ti}$, (**b**) ${\tilde{D}}_{ZrZr}^{Ti}$ as functions of composition at annealing temperatures of 1373 K, 1423 K, and 1473 K, cover Zr content from 0 to 0.16 and Ta content from 0 to 0.16.

Figure 7 shows the composition-dependent diffusion coefficients at diverse annealing temperatures, which were computed via the numerical inverse method coupled with HitDIC software for high-throughput analytical purposes. The scope of these calculations encompasses Zr contents ranging from 0 to 0.16 and Ta contents spanning 0 to 0.16. Three-dimensional surface plots corresponding to the principal diffusion coefficients ${\tilde{D}}_{TaTa}^{Ti}$ and ${\tilde{D}}_{ZrZr}^{Ti}$ are illustrated in Figure 7a, b. This figure reveals a dual dependence of two groups of three-dimensional diffusion planes on both composition and temperature. It indicates that with the increasing temperature, all diffusion coefficients exhibit an increasing trend, i.e., elevated temperatures accelerate the diffusion rate, in agreement with the results of the aforementioned composition–distance curves. Variations in elemental composition exert a significant influence on diffusion behavior: the main diffusion coefficient ${\tilde{D}}_{TaTa}^{Ti}$ decreases as Ta content increases, yet increases as Zr content increases, attaining its maximum value at the Ti–Zr composition. The main diffusion coefficient ${\tilde{D}}_{ZrZr}^{Ti}$ decreases primarily with the increase in Ta content and is essentially independent of the variation in Zr content. Specifically, the 95% confidence intervals for the maximum values of ${\tilde{D}}_{TaTa}^{Ti}$ at 1373 K, 1423 K, and 1473 K are (2.04 ± 0.1224) × 10^−13^ m^2^/s, (3.28 ± 0.1968) × 10^−13^ m^2^/s, and (5.13 ± 0.3078) × 10^−13^ m^2^/s, respectively. Under the same temperatures, the 95% confidence intervals for the maximum values of ${\tilde{D}}_{ZrZr}^{Ti}$ are (1.11 ± 0.0666) × 10^−12^ m^2^/s, (1.67 ± 0.1002) × 10^−12^ m^2^/s, and (2.46 ± 0.1476) × 10^−12^ m^2^/s. At equivalent temperatures, the main diffusion coefficient ${\tilde{D}}_{ZrZr}^{Ti}$ is one order of magnitude higher than ${\tilde{D}}_{TaTa}^{Ti}$. The obtained diffusion coefficients in this work are in good agreement with those previously reported for similar β-titanium alloys in the literature [20].

**Figure 8:**
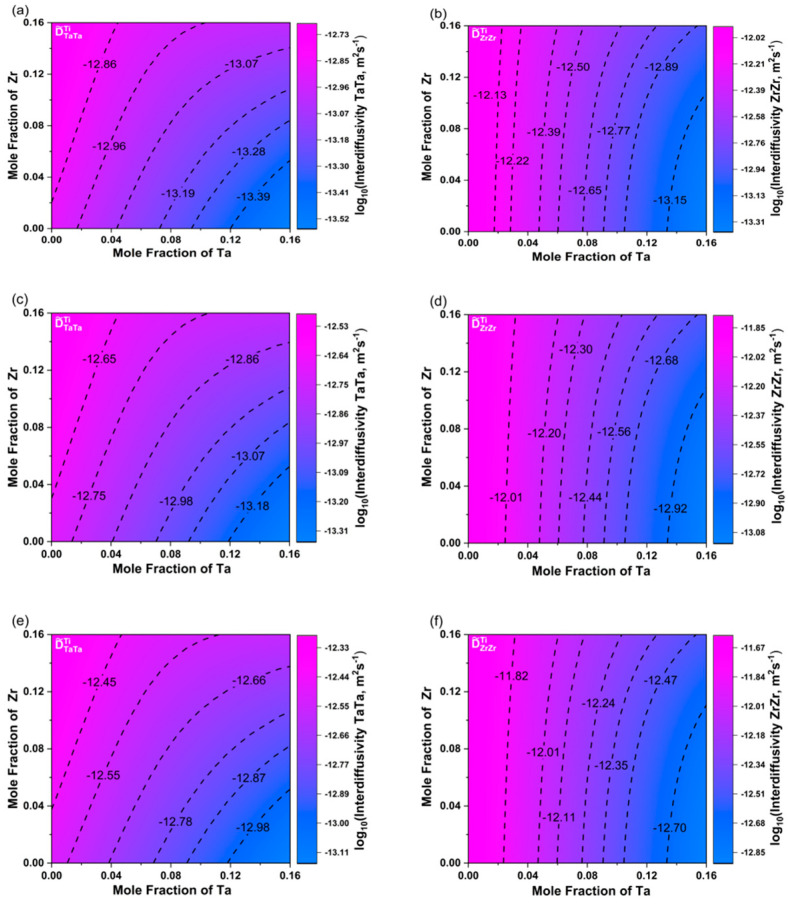
Contour plots at different temperatures: (**a**) ${log}_{10}^{{\tilde{D}}_{TaTa}^{Ti}}$ at 1373 K, (**b**) ${log}_{10}^{{\tilde{D}}_{ZrZr}^{Ti}}$ at 1373 K, (**c**) ${log}_{10}^{{\tilde{D}}_{TaTa}^{Ti}}$ at 1423 K, (**d**) ${log}_{10}^{{\tilde{D}}_{ZrZr}^{Ti}}$ at 1423 K, (**e**) ${log}_{10}^{{\tilde{D}}_{TaTa}^{Ti}}$ at 1473 K, (**f**) ${log}_{10}^{{\tilde{D}}_{ZrZr}^{Ti}}$ at 1473 K.

In order to more vividly reveal how key diffusion coefficients change with alloy composition, contour plots corresponding to the calculated key diffusion coefficients were drawn in this research under three distinct temperature conditions: 1373 K, 1423 K, and 1473 K. The contour plots reflecting the logarithmic values of two principal diffusion coefficients, ${\tilde{D}}_{TaTa}^{Ti}$ and ${\tilde{D}}_{ZrZr}^{Ti}$, are presented in Figure 8. It can be directly observed from the contour plots of ${\tilde{D}}_{TaTa}^{Ti}$ that the color of the contour lines changes more significantly with the variation in the Ta composition axis. This indicates that ${\tilde{D}}_{TaTa}^{Ti}$ is more dependent on Ta than Zr. In the contour plots of ${\tilde{D}}_{ZrZr}^{Ti}$, the color of the contour lines also changes more obviously along the Ta composition axis, with a much denser distribution. This demonstrates that ${\tilde{D}}_{ZrZr}^{Ti}$ is more dependent on Ta than on Zr, and its value varies primarily with the Ta content while being less affected by the variation in Zr content.

**Figure 9:**
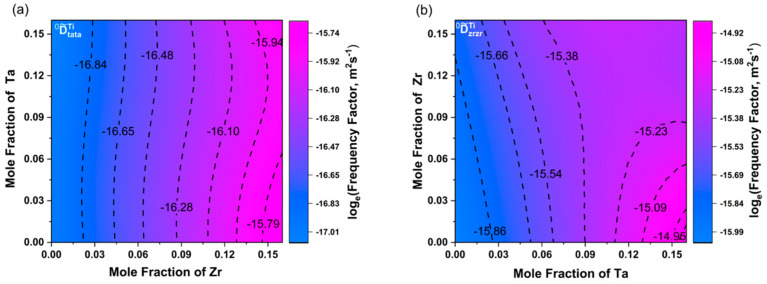
Contour plots of frequency factors: (**a**) D~TaTaTi0, (**b**) D~ZrZrTi0.

**Figure 10:**
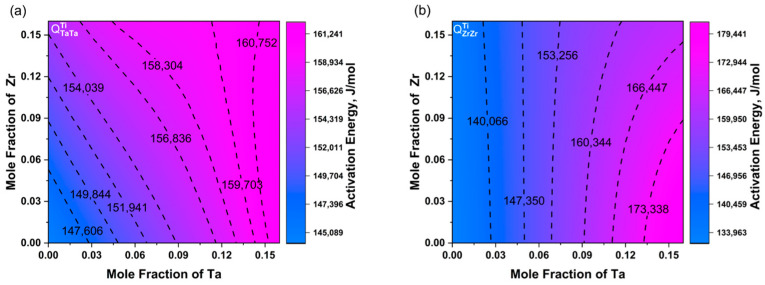
Contour plots of activation energies: (**a**) $Q_{TaTa}^{Ti}$, (**b**) $Q_{ZrZr}^{Ti}$.

To further understand diffusion behavior beyond temperature dependence, Arrhenius relationships of major components in the Ti–Zr–Ta system were investigated. Frequency factors and activation energies for respective diffusion mobilities were evaluated, as shown in Figure 9 and Figure 10. By utilizing the diffusion coefficients derived from the numerical inverse method under various annealing temperature conditions, the activation energies and pre-exponential factors corresponding to Zr and Ta components can be calculated through the application of the Arrhenius equation:

(16) D~iiTi =D~iiTi0exp(−Q/RT)

where ${\tilde{D}}_{ii}^{Ti}$ represents the diffusion coefficient (i = Zr or Ta), D~iiTi0 represents the frequency factor, Q represents the activation energy, R represents the gas constant, and T represents the temperature.

Figure 9 and Figure 10 show the contour plots of the frequency factor and activation energy, respectively. Figure 9a indicates that D~TaTaTi0 increases with increasing Zr content, and Figure 9b shows that D~ZrZrTi0 increases mainly with increasing Ta content. In Figure 10a,b, both $Q_{TaTa}^{Ti}$ and $Q_{ZrZr}^{Ti}$ increase with increasing Ta content.

The activation energy is closely related to some physical properties of Ti, Ta, and Zr, such as melting temperature Tm, atomic radius, and interatomic bonding strength. A higher melting temperature corresponds to stronger interatomic bonding and higher energy required for atomic diffusion. Therefore, Ta, which has a higher melting temperature than Zr, exhibits a larger activation energy. A larger atomic radius difference leads to more severe lattice distortion, which increases the diffusion resistance and thus the activation energy. The radius difference between Zr and Ta is larger than that between Zr and Ti, which explains why $Q_{TaTa}^{Ti}$ and $Q_{ZrZr}^{Ti}$ vary more significantly along the Ta composition axis, i.e., the lattice distortion caused by the variation in Ta content is more pronounced. Meanwhile, the interatomic bonding strength, governed by the electronegativity difference, further regulates the activation energy. The electronegativity difference between Ti and Ta is smaller than that between Ti and Zr, resulting in a stronger covalent character of Ti–Ta bonds. This stronger bonding raises the diffusion energy barrier, so the variation in Ta content exerts a more significant influence on $Q_{TaTa}^{Ti}$ and $Q_{ZrZr}^{Ti}$.

## 5. Conclusions

In this study, twelve groups of Ti–Zr–Ta single-phase diffusion couples with a BCC_A2 structure were successfully prepared by employing annealing temperatures of 1373 K, 1423 K, and 1473 K. Electron probe microanalysis was utilized to accurately determine the composition–distance curves. The main interdiffusion coefficients were efficiently calculated using the HitDIC software combined with the numerical inverse method, over the temperature range of 1373 K–1473 K and the composition ranges of 0–16 at. % Zr and 0–16 at. % Ta. The ternary atomic mobility parameters applicable to the β-phase region of the Ti–Zr–Ta system were also obtained. Meanwhile, the extrapolation results beyond the above-mentioned temperature and composition ranges have not been verified in the present study, which still entails certain limitations. The results obtained by the numerical inversion method are consistent with those calculated using the Matano–Kirkaldy method. Based on the Arrhenius equation, contour plots of frequency factor (^0^${\tilde{D}}_{TaTa}^{Ti}$, ^0^${\tilde{D}}_{ZrZr}^{Ti}$) and activation energy ($Q_{TaTa}^{Ti}\;$ and $Q_{ZrZr}^{Ti}$) were constructed, and the diffusion characteristics of the Ti–Zr–Ta system as well as their regulation mechanisms by composition and temperature were systematically analyzed. Since the derived activation energies rely on experimental data from only three temperatures (1373 K, 1423 K, and 1473 K), they carry certain uncertainties. Expanding the temperature range in future research will help improve the accuracy of activation energy calculations. The main diffusion coefficients increase significantly with the elevation of temperature. Among them, the values of ${\tilde{D}}_{TaTa}^{Ti}$ increases with the decrease in Ta content and the increase in Zr content, reaching the 95% confidence intervals for the maximum values at 1373 K, 1423 K, and 1473 K of (2.04 ± 0.1224) × 10^−13^ m^2^/s, (3.28 ± 0.1968) × 10^−13^ m^2^/s, and (5.13 ± 0.3078) × 10^−13^ m^2^/s, respectively. The values of ${\tilde{D}}_{ZrZr}^{Ti}$ exhibit a decreasing trend with the increase in Ta content, with the 95% confidence intervals for the maximum values at 1373 K, 1423 K, and 1473 K of (1.11 ± 0.0666) × 10^−12^ m^2^/s, (1.67 ± 0.1002) × 10^−12^ m^2^/s, and (2.46 ± 0.1476) × 10^−12^ m^2^/s. The main diffusion coefficient ${\tilde{D}}_{ZrZr}^{Ti}$ is an order of magnitude higher than ${\tilde{D}}_{TaTa}^{Ti}$ at the same temperature, meaning that Zr atoms have higher mobility than Ta.

## Figures and Tables

**Table 1:** Overview of end compositions, diffusion temperatures and diffusion times in BCC Ti–Zr–Ta diffusion couples.

Couple Name	Actual Composition (Mole %)	Temperature (K)	Diffusion Time (h)
A1	Ti-15Zr/Ti-12Ta	1373	48
A2	Ti-8Zr/Ti-8Ta	1373	48
A3	Ti/Ti-5Zr-15Ta	1373	48
A4	Ti/Ti-15Zr-5Ta	1373	48
B1	Ti-15Zr/Ti-12Ta	1423	36
B2	Ti-8Zr/Ti-8Ta	1423	36
B3	Ti/Ti-5Zr-15Ta	1423	36
B4	Ti/Ti-15Zr-5Ta	1423	36
C1	Ti-15Zr/Ti-12Ta	1473	24
C2	Ti-8Zr/Ti-8Ta	1473	24
C3	Ti/Ti-5Zr-15Ta	1473	24
C4	Ti/Ti-15Zr-5Ta	1473	24

**Table 2:** Atomic mobility parameters of BCC_A2 phase for Ti–Zr–Ta system.

Mobility	Parameters	Reference
Mobility of Ti	$\phi_{Ti}^{Ti}=$ RTln(5.91 × 10^−5^ exp(−237,000/R/T) + 1.47 × 10^−8^ exp(−121,000/R/T))	[19]
	$\phi_{Ti}^{Zr}=$ −140,356.5 − 138.1 × T	[20]
	$\phi_{Ti}^{Ta}=$ −438,280.83 − 88.17 × T	[21]
	$\phi_{Ti}^{Zr,Ti}=$ −30,430.63 + 66.44 × T	[20]
	$\phi_{Ti}^{Ta,Ti}=$ 254,673.89 − 79.1 × T	[21]
	$\phi_{Ti}^{Ta,Zr}=$ 298,144.53 + 299.71 × T	This work
Mobility of Zr	$\phi_{Zr}^{Zr}=$ RTln(2.8 × 10^−10^ exp(−9754.63/T) + 1.73 × 10^−6^ exp(−23,346.16/T))	[20]
	$\phi_{Zr}^{Ti}=$ −131,670.6 − 133.4 × T	[20]
	$\phi_{Zr}^{Ta}=$ −448,833 + RTln(1.24 × 10^−4^)	[22]
	$\phi_{Zr}^{Zr,Ti}=$ −22,865.39 + 35.42 × T	[20]
	$\phi_{Zr}^{Ta,Ti}=$ 3515.63 + 3.03 × T	This work
Mobility of Ta	$\phi_{Ta}^{Ta}$ = RTln(1.89 × 10^−7^ exp (−370,800/R/T) + 1.16 × 10^−4^ exp(−461,500/R/T))	[21]
	$\phi_{Ta}^{Zr}=$ −226,541 + RTln(7.29 × 10^−5^)	[22]
	$\phi_{Ta}^{Ti}=$ RTln(2.8 × 10^−8^ exp (−(140,300/R)/T) + 1.1 × 10^−3^ exp(−309,800/R/T))	[21]
	$\phi_{Ta}^{Ta,Ti}=$ 115,669.43 − 36.48 × T	[21]
	$\phi_{Ta}^{Zr,Ti}=$ 20,605.47 + 9.86 × T	This work

## Data Availability

The original contributions presented in the study are included in the article, further inquiries can be directed to the corresponding authors.
